# Parkin promotes proteasomal degradation of p62: implication of selective vulnerability of neuronal cells in the pathogenesis of Parkinson’s disease

**DOI:** 10.1007/s13238-015-0230-9

**Published:** 2016-01-08

**Authors:** Pingping Song, Shanshan Li, Hao Wu, Ruize Gao, Guanhua Rao, Dongmei Wang, Ziheng Chen, Biao Ma, Hongxia Wang, Nan Sui, Haiteng Deng, Zhuohua Zhang, Tieshan Tang, Zheng Tan, Zehan Han, Tieyuan Lu, Yushan Zhu, Quan Chen

**Affiliations:** State Key Laboratory of Medicinal Chemical Biology, Tianjin Key Laboratory of Protein Science, College of Life Sciences, Nankai University, Tianjin, 300071 China; State Key Laboratory of Biomembrane and Membrane Biotechnology, Institute of Zoology, Chinese Academy of Sciences, Beijing, 100101 China; Institute of Psychology, Chinese Academy of Sciences, Beijing, 100101 China; College of Life Sciences, Tsinghua University, Beijing, 100084 China; State Key Laboratory of Medical Genetics, Xiangya Medical School, Central South University, Changsha, 410078 China; Department of Health and Sports Science, Tianjin University of Sport, Tianjin, 300381 China

**Keywords:** parkin, sequestosome1/p62, ubiquitin, substantia nigra

## Abstract

Mutations or inactivation of *parkin*, an E3 ubiquitin ligase, are associated with familial form or sporadic Parkinson’s disease (PD), respectively, which manifested with the selective vulnerability of neuronal cells in substantia nigra (SN) and striatum (STR) regions. However, the underlying molecular mechanism linking parkin with the etiology of PD remains elusive. Here we report that p62, a critical regulator for protein quality control, inclusion body formation, selective autophagy and diverse signaling pathways, is a new substrate of parkin. P62 levels were increased in the SN and STR regions, but not in other brain regions in *parkin* knockout mice. Parkin directly interacts with and ubiquitinates p62 at the K13 to promote proteasomal degradation of p62 even in the absence of ATG5. Pathogenic mutations, knockdown of parkin or mutation of p62 at K13 prevented the degradation of p62. We further showed that parkin deficiency mice have pronounced loss of tyrosine hydroxylase positive neurons and have worse performance in motor test when treated with 6-hydroxydopamine hydrochloride in aged mice. These results suggest that, in addition to their critical role in regulating autophagy, p62 are subjected to parkin mediated proteasomal degradation and implicate that the dysregulation of parkin/p62 axis may involve in the selective vulnerability of neuronal cells during the onset of PD pathogenesis.

**Electronic supplementary material:**

The online version of this article (doi:10.1007/s13238-015-0230-9) contains supplementary material, which is available to authorized users.

## Introduction

Parkinson’s disease (PD) is one of the most common neurodegenerative diseases affecting over 2% of the population over 65 years of age. The selective loss of dopaminergic neurons that project from the midbrain substantia nigra (SN) to the striatum (STR) could account for the movement disorder symptom in PD (Ishikawa and Tsuji, 1996; Thomas and Beal, 2007). Sporadic PD or “classical parkinsonism” accounts for the majority of the disease and is multisystem neurodegenerative disorders, morphologically characterized by Lewy bodies (LBs) formation. The formation of LBs includes the stepwise condensation to ubiquitinated dense filamentous inclusions with incorporation of alpha-synuclein (Singleton et al., 2003; Spillantini et al., 1997) or p62 (Nakaso et al., 2004), which ultimately invoke the death and disappearance of the involved neurons, and ubiquitination seems to increase aggregation and neurotoxicity of alpha-synuclein in cultured human dopaminergic cells (Lee et al., 2008; Rott et al., 2008). A number of genes have been identified, and investigation of the underlying mechanisms of how these genes function has provided tremendous insights into the pathogenesis of both familial and sporadic PD (Bossy-Wetzel et al., 2004; Dawson, 2007; Dawson and Dawson, 2003; Farrer, 2006). In particular, mutations in parkin represent one of the major causes of early-onset familial PD (Biskup et al., 2008; Kitada et al., 1998; Lesage and Brice, 2009); It was thus proposed that mutations in parkin, an E3 ubiquitin ligase which ubiquitinates and degrades a diverse array of substrates, would cause accumulation and aggregation of these substrates due to insufficient E3 ligase activity for ubiquitin-proteasomal dependent protein turnover (Kahle and Haass, 2004; Li and Guo, 2009; Sriram et al., 2005). However, a few of known substrates were found to be accumulated in parkin deficient mice brain or in disease phenotypes, and the molecular link of how mutation of parkin leads to the etiology of PD remains elusive. Recent studies have revealed that parkin plays general role for mitochondrial motility and mitochondrial quality (Bingol et al., 2014; Gegg et al., 2010; Matsuda et al., 2010; Narendra et al., 2008; Tanaka et al., 2010) through modulating the stability of Miro (Wang et al., 2011b) and many other mitochondrial proteins (Chen and Dorn, 2013; Gegg and Schapira, 2011). It is thus proposed that mutation of parkin could results in mitochondrial dysfunction, which may causally link with the pathogenesis of PD. However, knockout of parkin in mice could not faithfully recapitulate the PD phenotype, raising the question of the physiological function and the pathologic role of parkin in PD (Dawson and Dawson, 2010; Johnson et al., 2012; Shin et al., 2011).

P62, also known as sequestosome 1, is a shuttle protein transporting polyubiquitinated proteins for both the proteasomal and autophagy/lysosomal dependent degradation (Komatsu et al., 2007; Pankiv et al., 2007; Seibenhener et al., 2004; Wooten et al., 2008). P62 and ubiquitinated proteins are conserved markers of neuronal aging, aggregate formation and progressive autophagic defects (Bartlett et al., 2011). In particular, p62 was commonly detected in ubiquitinated protein aggregates in neuronal diseases including LBs in PD, neurofibrillary tangles in Alzheimer’s disease, Huntington aggregates in Huntington’s disease, and skein-like inclusions in amyotrophic lateral sclerosis (Lowe et al., 1988; Rue et al., 2013; Seibenhener et al., 2004; Zatloukal et al., 2002). P62 shuttles misfolded proteins to the aggresome and autophagosome (Bjorkoy et al., 2006; Kirkin et al., 2009; Pankiv et al., 2010). Mutations in p62 have been linked with the occurrence of familial and sporadic amyotrophic lateral sclerosis (Fecto et al., 2011; Rubino et al., 2012). Furthermore, knockout of the p62 protein alone leads to neuropathological lesions including the accumulation of hyperphosphorylated tau and neurofibrillary tangles, synaptic deficiencies with loss of working memory and neuronal apoptosis (Babu et al., 2005; Wooten et al., 2008). It is thus crucial to maintain a homeostatic level of p62 for normal cellular functions. Dysregulation of p62 could result in the perturbation of cell signaling and accumulation of damaging protein aggregates, leading to neuronal loss and pathogenesis of neurodegenerative diseases. In an effort to understand whether and how parkin deficiency leads to the dysregulated mitochondrial dynamics and mitochondrial quality, we were interested to find that p62 is a new substrate of parkin and p62 is selectively accumulated in dopamingeric neuronal cells in parkin deficient mice. Our results showed that parkin plays a critical role for regulating p62 stability and implied that dysregulation of parkin/p62 axis could account for the selective vulnerability during pathogenesis of PD.

## Results

### P62 level is negatively correlated with parkin activity

**Figure 1:**
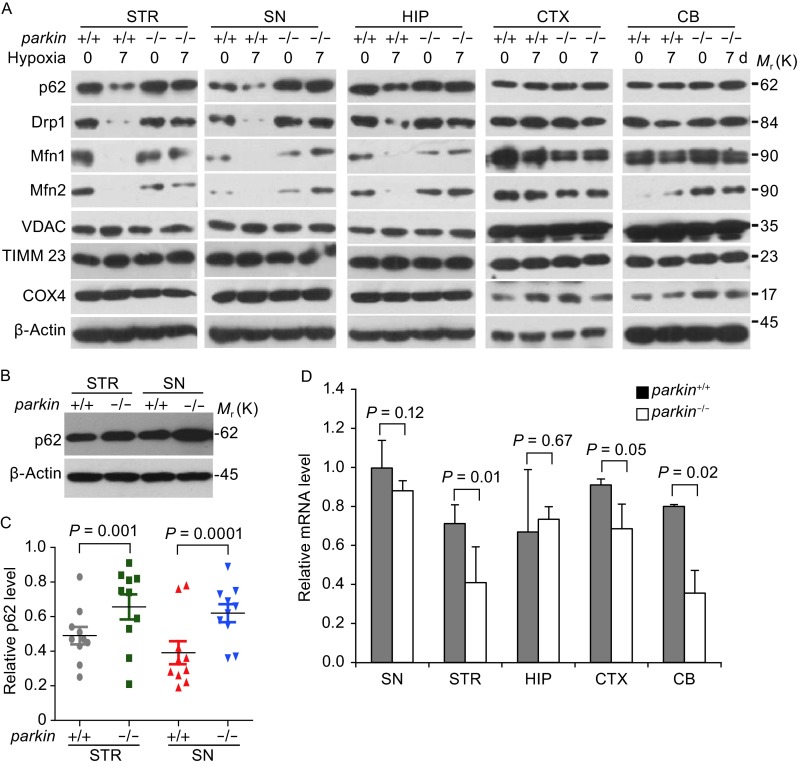
**P62 level is negatively correlated with parkin activity** ***in vivo***. (A) P62 is reduced in STR and SN of *parkin* ^+/+^, but not *parkin* ^−/−^ mice under hypoxic stress. *parkin* ^+*/*+^and *parkin* ^−/−^ mice, 18-month-old male C57Bl/6, were treated with 8% oxygen conditions for 0 (control) or 7 days. The striatum (STR), substantia nigra (SN), Hippocampus (HIP), frontal cortex (CTX) and cerebellum (CB) regions were isolated and homogenized in lysis buffer. Western blotting was performed to examine the level of indicated proteins. (B) P62 level increased in *parkin* ^−/−^ mice in STR and SN regions. The STR and SN regions from 8-week-old male C57Bl/6 mice brain were further isolated and homogenized in lysis buffer, and Western blotting was performed to examine the level of p62 (A). (C) Relative protein levels of p62 of individual mice in (1B) were quantified according to the results of ten independent blots and normalized to β-actin. (D) The mRNA levels detected by qPCR in mice tissues were described in Fig. 1B and 1D. The intensity of bands was measured with Image J software in B, mean ± SEM, from 3 independent experiments, one-way ANOVA, the *P*-value were indicated figures

Neuronal cells in the brain are highly sensitive to oxygen for energy production and neuronal activity. We thus were interested to measure the mitochondrial protein levels could change in response to hypoxic treatments *in vivo*. We first treated the mice for 7 days in 8% oxygen chamber and isolated different regions of brain, including the striatum (STR), substantia nigra (SN), hippocampus (HIP), frontal cortex (CTX) and cerebellum (CB), and then compared the protein levels of a number of mitochondrial and autophagy markers before and after hypoxic treatment. Mitochondrial proteins such as Mfn1/2, Drp1 in STR, HIP and SN were significantly reduced in wild-type mice, while other mitochondrial proteins such as VDAC1, TIMM23, COX4 were maintained. However, no changes of Drp1 and Mfn1/2 levels were observed in other regions including the cerebellum and the frontal cortex (Fig. 1A). Given Mfn1/2 and Drp1 are known substrates of parkin, we wondered that parkin may be involved in selectively degradation of Mfn1/2 and Drp1 in STR and SN regions and thus further compared the protein levels of a number of mitochondrial and autophagy markers in wild-type and germline *parkin* exon 3 knockout mice (*parkin*^−/−^) (Goldberg et al., 2003). The reduction of Mfn1/2, Drp1 protein levels in STR and SN regions were largely blocked in parkin deficient mice (Fig. 1A). Interestingly, we found that p62 levels were also reduced in STR, SN and HIP regions in response to hypoxia in wild-type mice, similar to the known mitochondrial substrates of parkin, while its protein levels were maintained in other brain regions and in parkin deficient mice (Fig. 1A). Careful examination of p62 levels revealed that there was an increase of p62 levels in the STR and SN of *parkin*^−/−^ mice brain compared to controls (Fig. 1B and 1C), while the other regions of the brain including the HIP, the CB and CTX exhibited no such increase (Fig. S1). Real-time PCR analysis showed that mRNA levels of p62 are reduced in the STR, CB and CTX, and there were no significant changes in the SN and HIP regions (Fig. 1D). These data demonstrate that parkin is involved in regulating mitochondrial protein levels in response to hypoxia in dopamingeric neuronal cells, and the protein levels of p62 are negatively correlated with the expression of parkin in the SN and STR regions that have selective vulnerability in PD.

### Parkin regulates p62 levels via a proteasomal-dependent pathway

**Figure 2:**
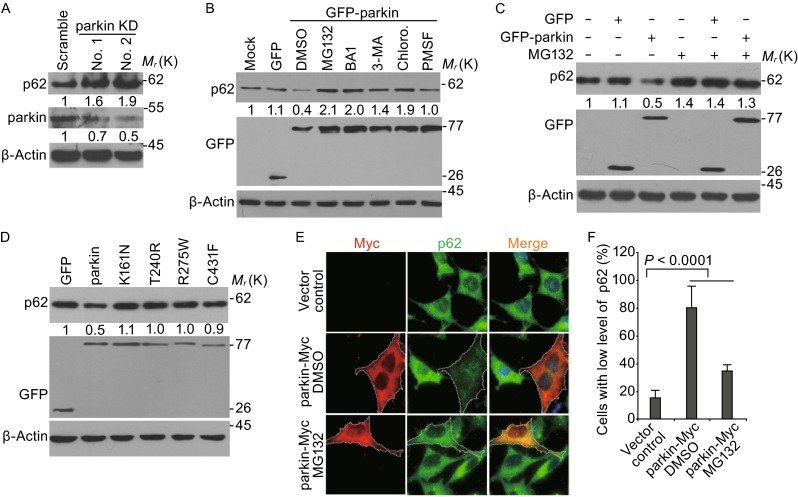
**Parkin mediates the degradation of p62 by both proteasomal and autophagic pathway**. (A) Knockdown of parkin increases p62 levels. Immunoblot analysis of parkin and p62 protein levels in SH-SY5Y cells transfected with shRNA specifically targeting two different regions of *parkin* mRNA. (B) Parkin-mediated p62 degradation can be inhibited by both proteasomal and autophagic inhibitors. SH-SY5Y cells were transfected with GFP or GFP-parkin for 24 h before treatment with inhibitors: MG132 (5 μmol/L), Bafilomycin A1 (20 nmol/L), 3-MA (10 mmol/L), Chloroquine (100 μmol/L), and PMSF (100 μmol/L) using DMSO as vehicle control. Cells were then harvested and immunoblotted with anti-p62 and anti-GFP antibodies. β-Actin was used as a loading control in the Western blotting analysis. (C) MG132 inhibited parkin induced p62 degradation. Cells were treated and analyzed as that in Fig. 2B in the presence or absence of 5 μmol/L MG132. (D) Wild-type, but not disease causing parkin mutants, reduces p62 levels. Cells were transfected with indicated plasmids for 24 h, then cell lysates were subjected to Western blotting by the anti-p62 and anti-GFP antibodies. (E) Immunostaining of Myc or parkin-Myc (red) and p62 (green) in SH-SY5Y cells transfected with Myc or parkin-Myc for 24 h. MG132 were added 8 h before fixed for assay the parkin and p62 protein levels. (F) Quantification of the cells with low level of p62 from Myc or parkin-Myc expression cells as shown in Fig. 2E. Mean ± SEM; *n* = 100 cells from 3 independent experiments, two-way ANOVA

**Figure 3:**
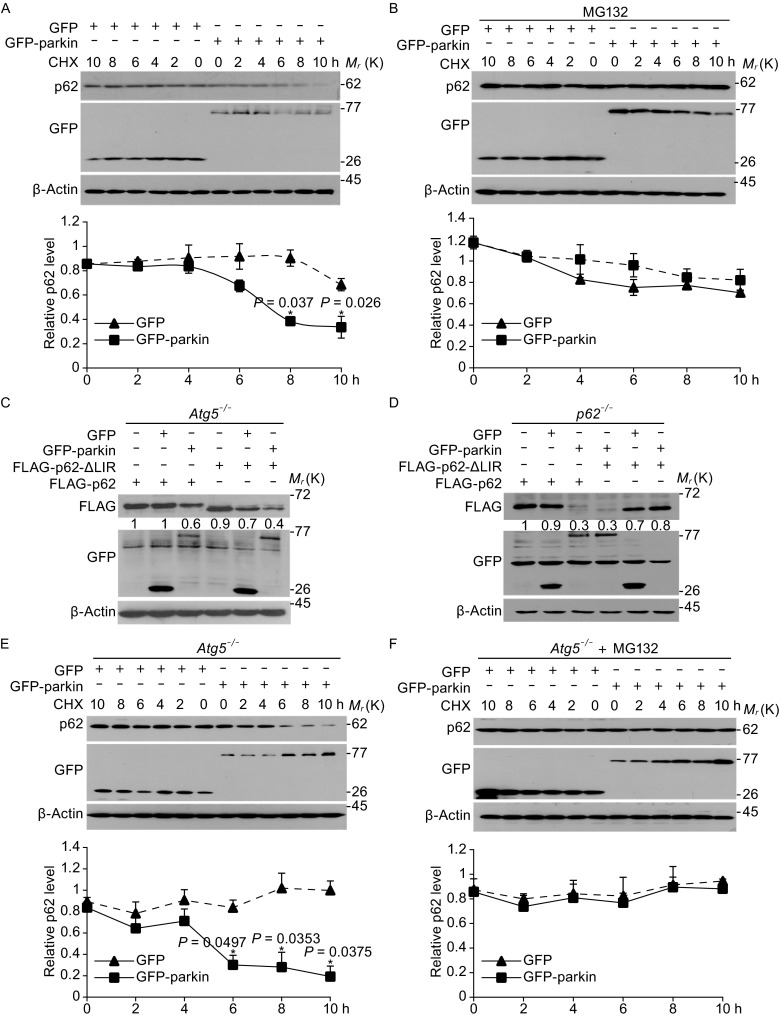
**Parkin mediated the degradation of** ***p62*** **in** ***Atg5*** ^**−/−**^ **MEF cells**. (A) Cycloheximide (CHX)-chase assay for the half-life of p62 in SH-SY5Y cells. Top panel, SH-SY5Y cells were transfected with GFP or GFP-parkin for 24 h, and then treated with CHX (100 μg/mL) for the indicated time, and Western blotting detected the indicated antibodies. Bottom panel, the level of remaining p62 at different time points was normalized to β-actin from 3 separate experiments. (B) Parkin decreases the steady-state levels of p62 in CHX-chase experiments in SH-SY5Y cells can be blocked by MG132. Top panel, SH-SY5Y cells were transfected with GFP or GFP-parkin for 24 h, and then treated with CHX (100 μg/mL) for the indicated time and 5 μmol/L MG132 for 8 h, and Western blotting detected the indicated antibodies. Bottom panel, the level of remaining p62 at different time points was normalized to β-actin and/or p62 levels at time 0 from 3 separate experiments. (C) P62 and its LIR mutant can be degraded in *Atg5* ^−/−^ MEF cells. *Atg5* ^−/−^ MEF cells were co-transfected with GFP or GFP-parkin and FLAG-p62 or FLAG-p62 LIR deletion mutant for 24 h. Cells were then harvested and Western blotted with anti-FLAG or anti-GFP antibodies. (D) P62 and its LIR mutant can be degraded in *p62* ^−/−^ MEF cells. *P62* ^−/−^ MEF cells were co-transfected with GFP or GFP-parkin and FLAG-p62 or FLAG-p62 LIR deletion mutant for 24 h. Cells were then harvested and Western blotted with anti-FLAG or anti-GFP antibodies. (E) CHX-chase assay for the half-life of p62 in *Atg5* ^−/−^ MEF cells. Top panel, *Atg5* ^−/−^ MEF cells were transfected with GFP or GFP-parkin for 24 h. Cells were then treated with CHX (100 μg/mL) for the indicated time, and Western blotting was performed with anti-p62 or anti-GFP antibodies. Bottom panel, the level of remaining p62 at different time points was normalized to p62 levels at time 0 from 3 separate experiments. (F) The decrease in the steady-state levels of p62 by parkin in CHX-chase experiments in *Atg5* ^−/−^ MEF cells can be blocked by MG132. Top panel, Cells was then treated with CHX (100 μg/mL) for the indicated time and 5 μmol/L MG132 for 8 h, and Western blotting was performed with anti-p62 or anti-GFP antibodies. Bottom panel, the level of remaining p62 at different time course was normalized to β-actin from 3 separate experiments. (The intensity of bands was measured with Image J software. mean ± SEM, from 3 independent experiments, one-way ANOVA, *P < 0.05 compared with control group)

Previous reports including ours have shown that parkin is a potent E3 ligase that mediates ubiquitination and proteasomal-dependent degradation of its substrates (Burchell et al., 2013; Ko et al., 2006; Sarraf et al., 2013; Wang et al., 2011a). We were thus prompted to understand if parkin promoted the proteasomal degradation of p62 in addition to its well-documented autophagic degradation. We first knocked down parkin by specific shRNA in SH-SY5Y cells, a neuroblastoma cell line that expresses endogenous parkin, and found that there was an accumulation of p62 when parkin was knocked down (Figs. 2A and S2). Conversely, overexpression of wild-type parkin significantly reduced the levels of p62, but not in those cells that expressed the vector alone (Fig. 2B–F). The disease-causing mutations in parkin with impaired E3 ligase activity (Sriram et al., 2005) failed to induce the reduction of p62 levels (Fig. 2D), indicating that the level of p62 is dependent on the E3 ligase activity of parkin. Consistent with previous reports (Ichimura and Komatsu, 2010; Komatsu and Ichimura, 2010), we found that the autophagic inhibitors Bafilomycin A1 (BA1), 3-MA and Chloroquine could also inhibit the reduction of p62 when wild-type parkin was expressed. MG132, a proteasomal inhibitor, could also potently inhibit p62 reduction when parkin is ectopically expressed (Fig. 2B and 2C). Immunofluorescent image analysis further confirmed that overexpression of parkin could reduce the levels of p62, which can be prevented by MG132 (Fig. 2E and 2F). To further substantiate this finding, we performed a cycloheximide (CHX)-chase assay and found a striking decrease of the p62 half-life in cells overexpressing GFP-parkin in SH-SY5Y cells, which can be inhibited by MG132 (Fig. 3A and 3B). Collectively, these data suggest that p62 levels can be down-regulated by both the autophagic and the proteasomal-dependent pathway.

To further ascertain the proteasomal-dependent degradation of p62 by parkin, the *Agt5*^−/−^ MEF cells (Fig. S3), in which autophagic activity is abrogated, were employed to detect the p62 protein. We transfected *Agt5*^−/−^ MEF cells with wild-type p62 or a LIR deletion mutant, which was reported to mediate its interaction with LC3 for autophagic degradation, and found that the protein level of p62 or its LIR deletion mutant were significantly reduced when parkin is expressed in these cells (Fig. 3C). The degradation of p62 and its LIR deletion mutant was also evident when p62 and parkin were expressed in *p62*^−/−^ MEF cells (Figs. 3D and S3). The CHX-chase assay further revealed a significant decrease of the p62 half-life in cells overexpressing GFP-parkin in *Atg5*^−/−^ MEF cells, which can be inhibited by MG132 (Fig. 3E and 3F). Taken together, we conclude that parkin functions as an E3 ligase to mediate the proteasomal degradation of p62, or in other words, p62 is a novel substrate of parkin.

### Parkin interacts with and ubiquitinates p62 for its degradation

**Figure 4:**
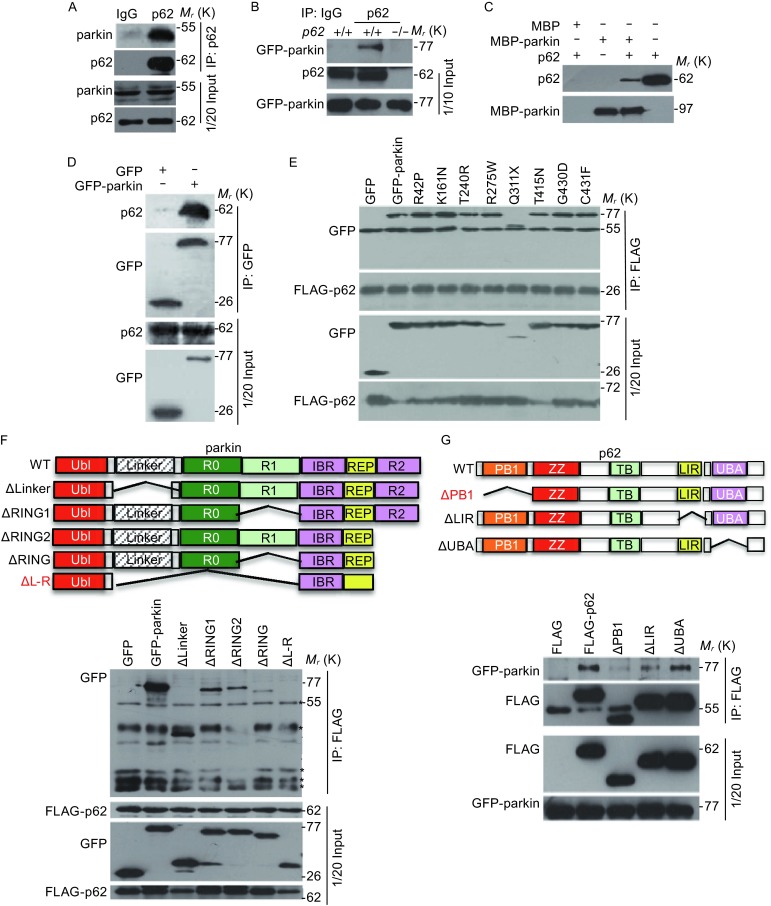
**Parkin interacts with p62** ***in vivo*** **and** ***in vitro***. (A) SH-SY5Y cells were harvested and the cell lysates were subjected to immunoprecipitation with an anti-p62 antibody or an IgG control, and the immunoprecipitates were examined by Western blotting using an anti-parkin antibody. (B) *P62* ^+/+^ or *p62* ^−/−^ MEF cells were transfected with GFP-parkin for 24 h, and cell lysates were subjected to immunoprecipitation with an anti-p62 antibody or an IgG control, and the immunoprecipitates were examined by Western blotting using anti-GFP or anti-p62 antibodies. (C) *In vitro* translated p62 was incubated with bacterially purified MBP-parkin or MBP immobilized on MBP beads, and Western blotting was performed to detect the p62 protein by using the anti-p62 antibodies. (D) 293T cells were transfected with GFP-parkin or GFP (control), and immunoprecipitation and Western blotting were performed to examine the interaction between GFP-parkin and endogenous p62. (E) 293T cells were co-transfected with FLAG-p62 and GFP-parkin or parkin mutants, and immunoprecipitation and Western blotting were performed to examine the interaction between p62 and parkin or parkin mutants. (F) Top panel, schematic representation of various deletion mutants of GFP-parkin, including the Linker domain deletion mutant, ΔLinker; RING1 deletion mutant, ΔRING1; RING2 deletion mutant, ΔRING2; RING1 and RING2 double deletion mutant, ΔRING; Linker and double RING finger deletion mutant, ΔL-R. Bottom panel, 293T cells were co-transfected with FLAG-p62 and GFP-parkin or parkin mutants, and immunoprecipitation and Western blotting were performed to examine the interaction between p62 and parkin. (G) Top panel, schematic representation of various deletion forms of FLAG-p62, including PB1 domain deletion mutant, ΔPB1; LIR deletion mutant, ΔLIR; UBA deletion mutant, ΔUBA. Bottom panel, 293T cells were co-transfected with GFP-parkin and FLAG-p62 or p62 deletion mutants, and immunoprecipitation and Western blotting were performed to examine the interaction between p62 and parkin

To understand the mechanisms of parkin mediated proteasomal degradation of p62, we first checked if these two molecules interact with each other. Co-immunoprecipitation analysis showed that endogenous p62 interacts with endogenous parkin (Fig. 4A and 4B). Pull-down analysis of recombinant MBP-parkin with recombinant p62 further showed that their interaction was direct (Fig. 4C). Ectopically expressed GFP-parkin, but not GFP itself, could interact with endogenous p62 (Fig. 4D). Although pathological mutations of parkin failed to induce the reduction of p62 levels, they still interact with p62 in cells (Fig. 4E). Domain mapping indicates that both the RING1 and RING2 domains, which mediate the E3 ligase activity, and the Linker domain of parkin were required for binding to p62 via its PB1 domain (Fig. 4F and 4G).

**Figure 5:**
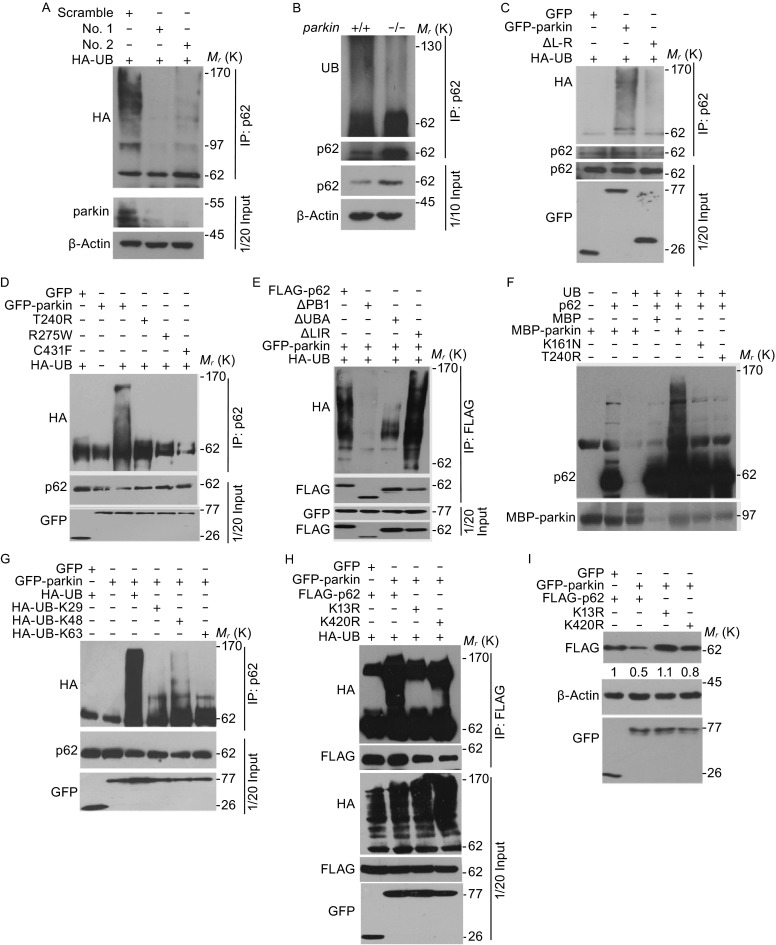
**Wild-type parkin, but not disease causing mutants, ubiquitinates p62**. (A) SH-SY5Y cells were transfected with HA-UB together with scramble or *parkin* siRNAs as indicated for 36 h. Cells were treated with 5 μmol/L MG132 for 8 h before harvest. Cell lysates were then subjected to immunoprecipitation and Western blotting to detect the ubiquitination of p62. (B) The midbrain of *parkin* ^+/+^ or *parkin* ^−/−^ mice, 8-week-old male C57Bl/6, were isolated and homogenized in lysis buffer. Cell lysates were then subjected to immunoprecipitation with anti-p62 antibody and Western blotting to detect the ubiquitination levels of p62. (C) SH-SY5Y cells were transfected with HA-UB together with GFP, GFP-parkin or ΔL-R for 36 h, and were treated with 5 μmol/L MG132 for 8 h before harvest. Cell lysates were then subjected to immunoprecipitation and Western blotting for the ubiquitination levels of p62. (D) SH-SY5Y cells were transfected with HA-UB together with GFP, GFP-parkin or parkin mutants (T240R, R275 W and C431F). Cells were treated with 5 μmol/L MG132 for 8 h before harvest. Cell lysates were then subjected to immunoprecipitation and Western blotting the ubiquitination levels of p62. (E) SH-SY5Y cells were transfected with HA-UB together with GFP-parkin and FLAG-p62 or p62 deletion mutants. Cells were treated with 5 μmol/L MG132 for 8 h before harvest. Cell lysates were then subjected to immunoprecipitation and Western blotting the ubiquitination levels of p62. (F) *In vitro* translated p62 protein was incubated with commercial purified ubiquitin, E1, E2 (UbcH7), and bacterially purified parkin proteins. The reaction products were analyzed by Western blotting with anti-p62 antibodies. (G) 293T cells were transfected with various HA-UB constructs (K29, K48 and K63) together with GFP-parkin or GFP vector. Cells were treated with 5 μmol/L MG132 for 8 h before harvest. Cell lysates were then subjected to immunoprecipitation and Western blotting the ubiquitination levels of p62. (H) 293T cells were transfected with HA-UB together with GFP-parkin and FLAG-p62 or mutants (K13R, K420R). Cells were treated with 5 μmol/L MG132 for 8 h before harvest. Cell lysates were then subjected to immunoprecipitation and Western blotting the ubiquitination levels of p62. (I) 293T cells were co-transfected with GFP or GFP-parkin and FLAG-p62 or K13R/K420R mutants for 24 h. Cells were then harvested and immunoblotted with anti-FLAG or anti-GFP antibodies. β-Actin was Western blotted as a loading control

We next examined whether parkin is able to ubiquitinate p62 for its subsequent degradation. Knockdown of parkin in SH-SY5Y cells by specific shRNA could significantly reduce the ubiquitination of p62 (Fig. 5A). Also, the level of ubiquitinated p62 was significantly higher in the midbrain of wild-type mice than that in *parkin*^−/−^ mice (Fig. 5B). P62 is ubiquitinated by wild-type parkin, but not by known disease-causing mutants (Fig. 5C and 5D). Furthermore, a Linker-RING finger deletion mutant parkin that fails to interact with p62 is also unable to mediate the ubiquitination of p62 (Fig. 5C). Similarly, the PB1 deletion mutant of p62, which does not interact with parkin, is not ubiquitinated, but not found in either UBA or LIR domain deletion (Fig. 5E). These data suggest that p62 is an authentic substrate of parkin in both cell and animal model systems.

### P62 is ubiquitinated at K13 site for proteasomal degradation

To directly confirm that p62 is directly ubiquitinated by parkin, we carried out *in vitro* ubiquitination assay and found that *in vitro* purified parkin ubiquitinates p62 in the presence of E1, E2, ubiquitin and ATP. These data demonstrate that the ubiquitination of p62 is specifically mediated by parkin, while the disease causing mutants that have impaired E3 ligase activity fail to ubiquiniate p62 for its subsequent degradation (Fig. 5F). Immunoprecipitation analysis revealed that parkin was able to induce the poly-ubiquitination of p62 in the presence of wild-type or K48 ubiquitin, but significantly reduced in the presence of K29 or K63 ubiquitin (Fig. 5G). This experimental result suggests that parkin mediates the poly-ubiuqitination of p62 mainly via K48-linked ubiquitin chains for proteasomal degradation, while K63 ubiquitin modification occurs to a lesser extent (Fig. 5G).

To further demonstrate that parkin ubiquitinates p62, we sought to determine the unique site of ubiquitination of p62 by parkin. We transfected 293T cells with HA-Ubiquitin (HA-UB), GFP-parkin and FLAG-p62 and immunoprecipitated with anti-FLAG antibody, and immunoprecipitates were further analyzed by mass spectrometry, the mass results showed that both K13 and K420 are ubiquitinated. To confirm that these K13 and K420 residues were the sites of ubiquitination by parkin, we mutated K13 or K420 to arginine and co-transfected these mutants with GFP-parkin in 293T cells. We found that both wild-type p62 and the p62 K420R mutant, but not the p62 K13R mutant, are ubiquitinated by parkin (Fig. 5H). Importantly, we showed that the protein levels of the p62 K13R mutant, but not wild-type p62 or the p62 K420R mutant, were not reduced in the presence of wild-type parkin (Fig. 5I).

### Parkin regulates p62 degradation in response to 6-OHDA

**Figure 6:**
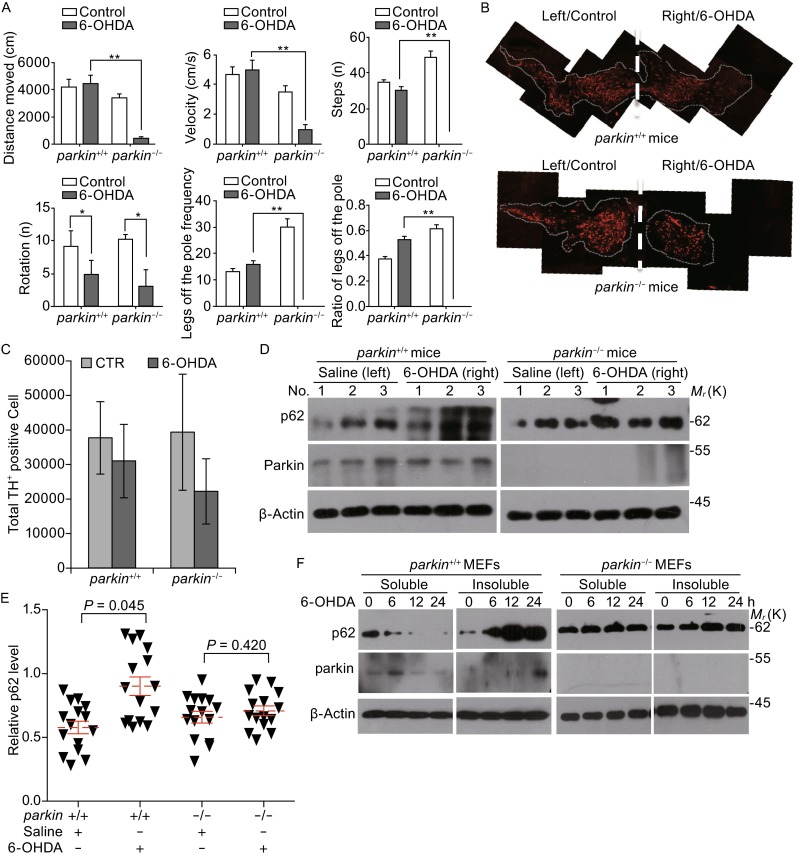
**Parkin affects p62 degradation in response to 6-OHDA treatments**. (A) The *parkin* ^+*/*+^and *parkin* ^−/−^ mice, 18-month-old male C57Bl/6 mice were injured by striatal stereotactic injections with 5.4 μg of 6-OHDA or saline after two weeks, behavioral tests were carried as described in MATERIALS AND METHODS. (B) The *parkin* ^+*/*+^ and *parkin* ^−/−^ mice, 18-month-old male C57Bl/6 mice were injured by striatal stereotactic injections from one side in brain with 5.4 μg of 6-OHDA or saline after two weeks, and the SN cells number was detected by immunohistochemical staining using anti-TH antibody antibodies. (C) Analysis of the SN cell number by Image-J soft was described in (B). (D) The mice described in A were sacrificed and the SN regions were isolated, then tissue lysate was subjected to Western blotting for the indicated protein levels. (E) The relative p62 protein levels were described in (D), which were normalized to β-actin from 3 separate experiments. (F) The *parkin* ^+*/*+^ and *parkin* ^−/−^ MEF cells were treated with 100 μmol/L 6-OHDA for indicated times, both the soluble and insoluble lysate were subjected to Western blotting for the indicated protein levels. All data are from three independent experiments (*n* = 7–9 mice). Mean ± SEM, one-way ANOVA, *P < 0.05, **P < 0.01 compared with control group

Consistent with previous reports, parkin deficient mice did not exhibit degeneration of dopaminergic neurons (Goldberg et al., 2003; Itier et al., 2003; Perez et al., 2005; Perez and Palmiter, 2005), likely due to the lack of aging related stresses. Dopamine can covalently modify and inactivate parkin through its conjugation with cysteine (431) (Lazarou et al., 2013) at its reactive center or making it becoming insoluble that diminishes its activity. As 6-OHDA is widely used to induce parkinsonal phenotypes in mice, we tested the functional implication of parkin for PD after 6-OHDA treatments. Consistent with previous reports (Perez and Palmiter, 2005), rotation and slip/step analysis do not reveal PD-like phenotypes in younger mice (6 months) (Fig. S5). However, such analysis showed that *parkin*^−/−^ aged mice (18 months) performed worse than that of wild-type controls upon injection of 6-OHDA (Fig. 6A). We also observed that there was pronounced loss of TH positive neurons in parkin deficient mice compared to its wild-type control after injections (Fig. 6B and 6C). These data suggest that parkin is of functional importance for selective loss of dopamingeric neurons and the onset of PD in aged mice. We further tested the effects of 6-OHDA on p62 and mitochondrial protein degradation *in vivo*. We directly injected 6-OHDA into SN region and examined the p62 protein levels and found that p62 levels are increased in wild-type mice, while the levels were maintained after the treatment with 6-OHDA (Fig. 6D and 6E). As only small amount of sample can be obtained from the SN regions of treated mice, we turned to analyze p62 degradation in response to 6-OHDA treatments in cultured cells. Indeed, treatment of 6-OHDA reduced the protein levels of parkin (likely due to its self-degradation) and such treatment significantly increased the p62 levels in the insoluble fractions in wild-type cells (Fig. 6F). And we also found the increase of p62 protein levels in *parkin*^−/−^ MEF cells compared with the wild type MEF cells (Data not shown).

## Discussion

The major findings in our current study are the identification of p62 as a new substrate of parkin and inactivation of parkin (either by genetic manipulations or enhanced degradation) may lead to the accumulation of p62 in dopamingeric neuronal cells and the selective vulnerability of these cells during the onset of the diseases. It is known that parkin is normally kept in the inactive state and can be either activated or inactivated through post-translational modification in response to mitochondrial or oxidative stresses. Once activated, parkin interacts with and subsequently ubiquitinates p62 at the K13 residue, resulting in the degradation of p62 via the proteasomal-dependent pathway. We found that the degradation of p62 can be blocked by the proteasomal inhibitor-MG132 even in *Atg5*^−/−^ MEF cells (Fig. 3). We clearly showed that p62 is required to be ubiquitinated by wild-type parkin at the K13 residue and mutation at this site blocked its degradation (Fig. 5). Recent work by Lee and colleagues suggested that p62 degradation could be inhibited by the proteasomal inhibitor MG132, which may also block the autophagic activities (Lee et al., 2012). Our results suggest that both proteasomal degradation and autophagic degradation are involved in p62 stability since BA1 and other lysosomal inhibitors also prevented its degradation (Fig. 2B). Their relative importance for p62 stability is likely cellular context and stress type dependent. For instance, mild stress such as hypoxia may promote p62 degradation in parkin dependent manner (Fig. 1A), while 6-OHDA induced parkin inactivation, p62 accumulation and aggregation and autophagic degradation (Fig. 6). The exact detail of how hypoxia activates parkin dependent p62 degradation requires further investigation and we observed the enhanced ubiquitination of p62 when treated with hypoxia, which is absent in *parkin*^−/−^ neuronal cells. Collectively, our results suggest a new link between these two important molecules that play essential roles for protein quality control, mitochondrial dynamics and cell signaling.

Both parkin and p62 were found to play role in mitophagy and/or general autophagy. In response to the loss of mitochondrial membrane potential, PINK1 becomes stabilized at the outer mitochondrial membrane where it can interact and phosphorylate parkin for its recruitments and activation at the site of mitochondria (Kane et al., 2014; Kazlauskaite et al., 2014; Koyano et al., 2014; Lazarou et al., 2015; Matsuda et al., 2010; Pickrell and Youle, 2015). Parkin then ubiquitinates a number of mitochondrial membrane proteins, both p62 and ULK1 are recruited toward mitochondria for selective cargo recognition (Li et al., 2015). For example, it is suggested that p62 can interact with LC3 for selective cargo recognition for mitophagy, although the exact role of p62 in parkin mediated mitophagy remains controversial. Much of the studies are carried out in the cultured cell system and physiological relevance of this critical mitophagy pathway with Parkinson’s diseases needs to be critically evaluated. Our results suggest that one of the physiological functions of parkin is to monitor the protein quality of its substrates through proteasomal degradation, while its mitophagic or autophagic role occurs under stress conditions such as protein aggregation or complete loss of mitochondrial membrane potential (Sterky et al., 2011). Previous studies have shown that parkin may not translocate onto mitochondria in response to the loss of mitochondrial membrane potential in primary neuronal cells (Van Laar et al., 2011) and is dispensable for the progressive mitochondrial respiration deficiency and loss of dopamine neuron caused by the loss of mtDNA (Sterky et al., 2011). It is possible that, during normal physiological situations, the parkin/p62 axis is able to keep cellular p62 levels in check for the well-being of the cell. As both parkin and p62 are sensitive to oxidative stress and the perturbation of redox signaling (Jain et al., 2010; LaVoie et al., 2007), it is possible that, when parkin is mutated or its E3 ligase activity is inhibited upon oxidative stress, there will be an increase in the level of p62. This increased level of p62 will initially be protective to the cells for the removal of misfolded proteins and protein aggregate through enhanced protein turnover or selective autophagy. However, drastic and persistent perturbation of the parkin/p62 axis, would redefine a threshold where proteins fail to degrade, neuronal signaling is impaired, and the hallmarks of PD are manifested.

P62 is a multifunctional protein involved in multiple cellular functions such as signal transduction and the degradation of both proteins and organelles. Accumulating protein aggregates, dysfunctional mitochondria and DNA damage contribute to the aging process and the onset of age-related conditions. Alzheimer’s (AD), Parkinson’s (PD), Huntington’s (HD) and other neurodegenerative diseases are characterized by the accumulation of protein aggregates in the brain. A different aggregation-prone protein characterizes the pathology of each of these diseases, but virtually all these protein aggregates associate with p62/SQSTM1. Our results thus uncover a potential role of parkin-p62 axis for the selective vulnerability of dopamingeric neuronal cells during the onset of the diseases. It is interesting that p62 levels are increased in STR and SN regions in *parkin*^−/−^ mice, while p62 in the other regions are not (Fig. 1). It was reported that there is a selective inactivation of parkin in the SN and STR in sporadic PD through nitrosative and dopaminergic stress in aged mice (Chung et al., 2004; LaVoie et al., 2005, 2007). It is thus conceivable that the selective inactivation of parkin in dopamingeric neurons may result in the accumulation and aggregation of p62 leading to the inclusion body formation (Komatsu et al., 2007) and toxic protein aggregation, critical step for Lewy body formation. We also noticed that aged mice showed the characteristic phenotype of movement disorder (Fig. S4), which younger mice have less obvious phenotypes, suggesting the aged related cellular events, such as protein oxidation and aggregation, or altered dopamine metabolisms may be involved (Goldberg et al., 2003), and our data directly showed that parkin mediated the aggregated p62 degradation in cells (Fig. S5). During aging, the inactivation of parkin in dopaminogeric neurons may promote the aggregation of p62 and neurotoxic proteins for the loss neuronal cells. Indeed, we observed the increase of insoluble p62 in the brain of PD patients (data not shown). Thus, our results are consistent with previous suggestions that inactivation of parkin is closely associated with the sporadic and progressive nature of PD. Further dissection of how the dysregulated parkin/p62 axis in dopamigeric neuronal cells will offer new insights of the molecular pathogenesis of PD and possible new intervention strategies for fighting PD.

## Materials and methods

### Cell cultures and plasmids

SH-SY5Y, 293T cells were cultured at 37°C (5% CO_2_) in DMEM (GIBCO) supplemented with 10% FBS (HyClone). The mammalian expression plasmids for GFP-parkin, HA-UB were generated as described previously (Wang et al., 2011a). The site mutants and deletion mutants of parkin were generated by PCR with different primers using pEGFPC1-parkin as template. Full length p62 cDNA was cloned into the pCMV-tag-2B vector. The deletion mutants of p62 were generated by PCR with different primers using pCMV-tag-2B-p62 as a template.

### Reagents and antibodies

Antibodies against Myc (Sc-40), GFP (Sc-9996) and HA (Sc-7392) were purchased from Santa Cruz. Antibodies against p62 (MBL PM045), β-actin (Sigma A5441), ATG5 (Sigma, A0856), parkin (Cell Signaling 2132, Millipore AB9244), FLAG (Sigma F1804) were from the indicated sources.

### Transfections and shRNAs

DNA transfections were performed using PEI according to the manufacturer’s instructions. The target sequences of parkin shRNA 1 and parkin shRNA 2 were 5′-gatccGTGATTTGCTTAGACTGTTTTTCAAGAGAAAACAGTCTAAGCAAATCATTTTTTg-3′, 5′-aattcAAAAAATGATTTGCTTAGACTGTTTTCTCTTGAAAAACAGTCTAAGCAAATCACg-3′; and 5′-gatccGCTTGGCTACTCCCTGCCTTTTCAAGAGAAAGGCAGGGAGTAGCCAAGTTTTTTg-3′, 5′-aattcAAAAAACTTGGCTACTCCCTGCCTTTCTCTTGAAAAGGCAGGGAGTAGCCAAGCg-3′.

The qPCR primers were as follows: p62-1, CAGAGAATACCTTTGCCTCCCA; p62-2, AATCTTGGAGCTCCCCATGTC; parkin-1, ATTCAGAAGCAGCCAGAGGTC; parkin-2, CTGGCACTCACCACTCATCC.

### Immunofluorescence analysis

SH-SY5Y cells transfected with parkin-Myc or Myc-vector grown on coverslips were washed with phosphate-buffered saline (PBS) and fixed in 4% formaldehyde in DMEM for 30 min at 37°C. Fixed cells were permeabilized with 0.2% Triton X-100 in PBS for 5 min at 4°C, and blocked with 3% BSA in PBS for 1 h. Then cells were stained with primary antibody (mouse anti-Myc, diluted 1:200; rabbit anti-p62, diluted 1:700) overnight at 4°C. After washing, cells were incubated with fluorescein isothiocyanate (FITC)-conjugated anti-rabbit IgG and Cy^tm^3-linked anti-mouse IgG for 1 h. Unbound antibody was removed with PBS, and cells were imaged using a Zeiss fluorescence microscope.

### Western blotting, immunoprecipitation and MBP pull-down

Western blotting, immunoprecipitation and MBP pull-down were performed, as described previously (Wang et al., 2011a). Briefly, cells were transfected with parkin or parkin mutant constructs for 36 h. Cells were washed with ice-cold PBS and lysed with lysis buffer (pNAS buffer: 50 mmol/L Tris–HCl [pH 7.5], 150 mmol/L NaCl, 1 mmol/L EDTA, and 1% Nonidet P-40). The soluble fractions were resolved by SDS-PAGE and transferred onto nitrocellulose filter membrane. Blots were probed with antibodies against p62, GFP, and β-actin. For immunoprecipitation, cell lysate was incubated with anti-FLAG antibody and then protein A/G-agarose beads (Pierce Biotechnology). The beads were washed extensively and boiled in SDS loading buffer, and the precipitated proteins were detected by Western blotting. For MBP pull-down, MBP or MBP-parkin fusion protein immobilized on amylose magnetic beads was incubated with *in vitro*-translated p62. The beads were washed and boiled in the SDS loading buffer, and the precipitated proteins were detected by Western blotting.

The soluble and insoluble cell lysate were prepared in buffer 1% Trion-100 and 1% SDS buffer described in reference (Kawahara et al., 2008).

### Ubiquitination assays

293T or SH-SY5Y cells were transfected with indicated tagged constructs in each experiment employing PEI. Cells were treated with 5 μmol/L MG132 for 8 h before harvesting. The cells were lysed for 30 min at 4°C in either pNAS buffer: 50 mmol/L Tris–HCl [pH 7.5], 150 mmol/L NaCl, 1 mmol/L EDTA, and 1% Nonidet P-40 (to detect noncovalent interaction) or 50 mmol/L Tris [pH 8.0], 150 mmol/L NaCl, 1% Triton, 0.5% sodium deoxycholate, and 0.1% sodium dodecyl sulfate (SDS) (to detect covalent interaction), both containing protease inhibitors. Cell lysate was incubated with anti-p62 antibody. The precipitates were subjected to Western blotting with anti-HA or anti-UB antibodies.

An *in vitro* ubiquitination assay was performed, as described previously (Wang et al., 2011a). Briefly, 2 μg MBP, MBP-parkin or MBP-parkin mutants, expressed and purified in a *E. coli* expression system, was incubated with *in vitro* translated p62 (2 μg) in 50 μL ubiquitintion reaction buffer, containing 50 mmol/L Tris–HCl [pH 7.5], 5 mmol/L MgCl_2_, 2 mmol/L DTT, 2 mmol/L ATP, 10 μg ubiquitin, 100 ng E1, and 200 ng E2 (UbcH7). Reaction was performed for 2 h at 25°C and terminated by addition of the SDS loading buffer. The reaction products were then subjected to Western blotting with anti-p62 antibodies.

### Immunocytochemical and histochemicalanalysis

Mice brains were removed and washed with ice-cold PBS. The brains then were post-fixed with 4% paraformaldehyde for 12 h and cryoprotected in 30% sucrose. Coronal sections were cut throughout the midbrain and sections were reacted with rabbit polyclonal anti-p62 and mouse monoclonal anti-Tyrosine hydroxylase (TH) and visualized with fluorescein isothiocyanate (FITC)-conjugated anti-rabbit IgG and cy^tm^3-linked anti-mouse IgG.

Four different brain regions from wild-type or *parkin* knockout mice were homogenized in lysis buffer containing 10 mmol/L Tris–HCl [pH 7.4], 150 mmol/L NaCl, 5 mmol/L EDTA, 0.5% Nonidet P-40, Phosphate Inhibitor Cocktail I and II (Sigma), and Complete Protease Inhibitor Mixture (Roche), using homogenizer. After homogenization, samples were rotated at 4°C for 30 min for complete lysis, then the homogenate was centrifuged at 14,000 rpm for 20 min, and the resulting fractions were collected and analyzed by immunoblot. Immunoblotting was performed with an antibody of interest and was performed with chemiluminescence (Pierce). The densitometric analyses of the bands were performed using Image-J. Data are expressed as mean ± SEM. The results were evaluated for statistical significance by applying the unpaired two-tailed Student’s *t* test.

### Mass spectrometry analysis of ubiquitination sites

293T cells were co-transfected with GFP-parkin and FLAG-p62. Cells were treated with 5 μmol/L MG132 for 8 h before harvest. Cell lysates were then subjected to immunoprecipitation with anti-FLAG antibody. The immunoprecipitates were resolved by SDS-PAGE and visualized by Coomassie blue staining. Protein samples were reduced, alkylated and digested with trypsin. The digests were subsequently analyzed by liquid chromatography tandem mass spectrometry.

### Chronic hypoxia treatments of mice

*parkin*^+/+^ or *parkin*^−/−^ mice (provided by Prof. Zhuohua Zhang from Central South University) were subjected to a mice hypoxic chamber for the indicated duration, with an atmosphere of 8% O_2_ in nitrogen and free access to food and water.

### 6-OHDA-lesion model of Parkinson’s disease in mice

Mice were anaesthetized using Chloral hydrate and placed into a stereotactic frame with nose and ear bars specially adapted for mice. 6-OHDA was dissolved at a concentration of 3 μg/μL saline in 0.1% ascorbic acid and injected at final dosages 5.4 μg. The lesion was performed using a Hamilton syringe at the following coordinates: AP: −2.9 mm; ML: +1.3 mm; DV: −4.6 mm. The injection was conducted at a rate of 0.2 μL/min and the needle was left in place for another 5 min after the injection before it was slowly drawn back. Wound healing and recovery were monitored after the injection was done.

### Animal behavior tests

The methods for the behavioral tests were described previously (Goldberg et al., 2003).

All animal tests were carried out between 9:00 and 15:00 and they were scored by the same rater in an observation sound-attenuated room under low-intensity light (12 l×), where the mice had been habituated for at least 1–2 h before the beginning of the tests. Behavior was monitored through a video camera positioned above the apparatuses and the videos were later analyzed. The apparatus were cleaned with 10% ethanol between animals to avoid odor cues. Briefly a Plexiglass beam consisting of four Sects. (25 cm each, 1 m total length) of varying width (3.5, 2.5, 1.5, and 0.5 cm) was used. Individual *parkin*^+/+^ or *parkin*^−/−^ mice were tested after being trained twice, and their performance was videotaped. The numbers of steps and slips were counted by viewing the videotapes in slow motion.

### Statistical analysis

Statistical analysis between groups was performed by unpaired two-tailed Student’s *t* test. Data are presented as mean ± SEM.

## Electronic supplementary material

Supplementary material 1 (PDF 1811 kb)
