# Supplementary material for: Parkin promotes proteasomal degradation of p62: implication of selective vulnerability of neuronal cells in the pathogenesis of Parkinson’s disease
Source: Protein Cell. 2016 Jan 8;7(2):114–29. doi: 10.1007/s13238-015-0230-9 (PMC4742389; doi:10.1007/s13238-015-0230-9)
Supplement: Supplementary file 1 — Supplementary material 1 (PDF 1811 kb) [file 13238_2015_230_MOESM1_ESM.pdf]

Figure S1

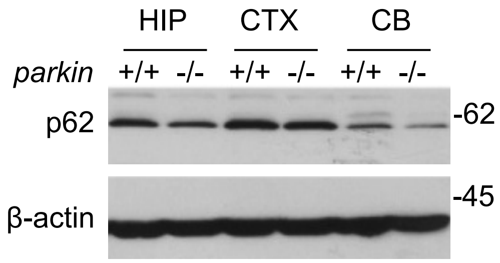

Figure S1. Detection the p62 levels in *parkin*$^{-/-}$ mice in vivo

The HIP, CTX and CB regions from 8-week-old male C57Bl/6 mice brain were further isolated and homogenized in lysis buffer, and Western blotting was performed to examine the level of p62.

Figure S2

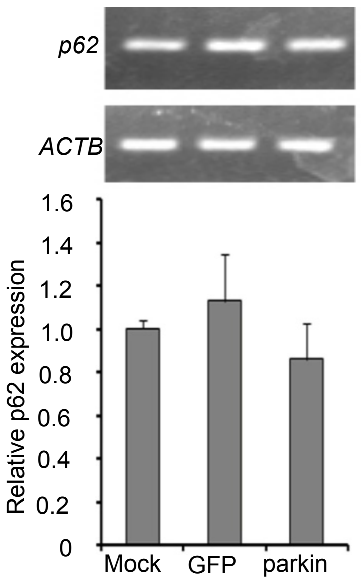

Figure S2. overexpression parkin does not affect the p62 mRNA level. The GFP-parkin or GFP were transfected into HeLa cells for 24 h, then PCR detected the mRNA levels for three times;

Figure S3

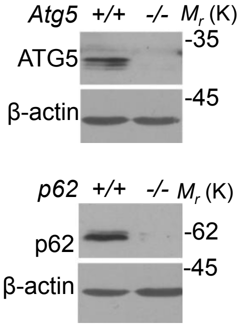

Figure S3. Detection of the ATG5 or p62 levels in Atg5 or p62 KO MEF cells.
The cells were collected and western blotting detected the ATG5 or p62 levels

Figure S4

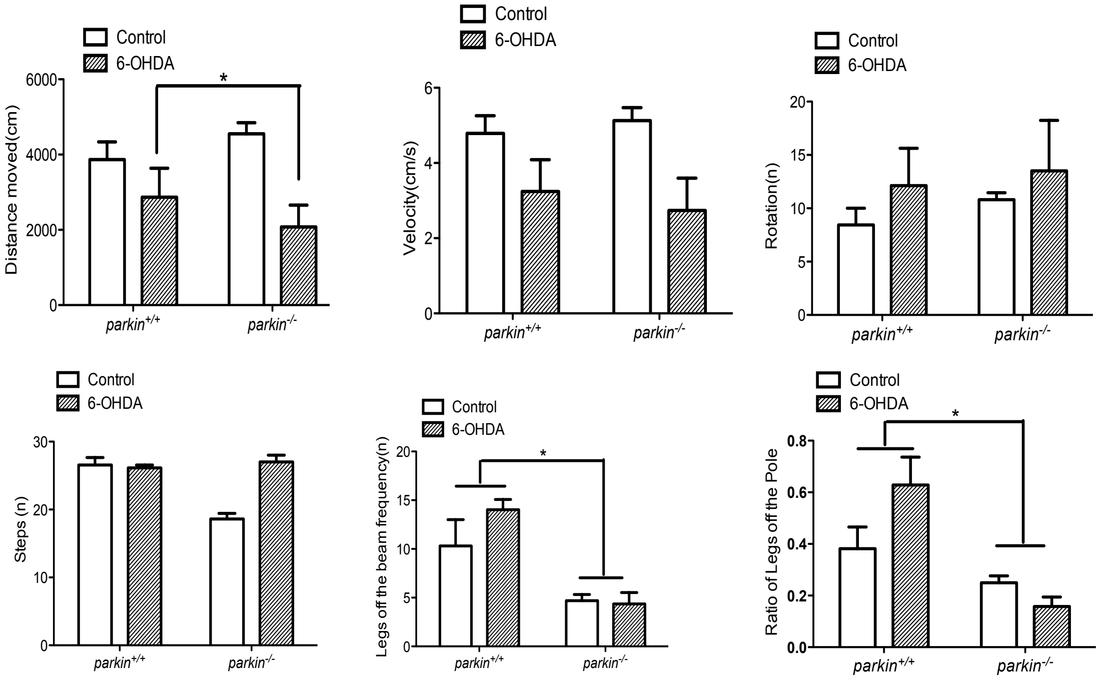

Figure S4. The behavioral tests were performed in 6 months-old mice.
The *parkin*$^{+/+}$and *parkin*$^{-/-}$ mice, 6 months-old male C57Bl/6 mice were lesioned by striatal stereotactic injections with 5.4 µg of 6-OHDA after two weeks, behavioral tests were carried.
All data are from three independent experiments (n = 10 mice). All error bars indicate SEM. **P* <*0.05,* Significance is determined by one-ANOVA .

Figure S5

Parkin degraded polymerized p62

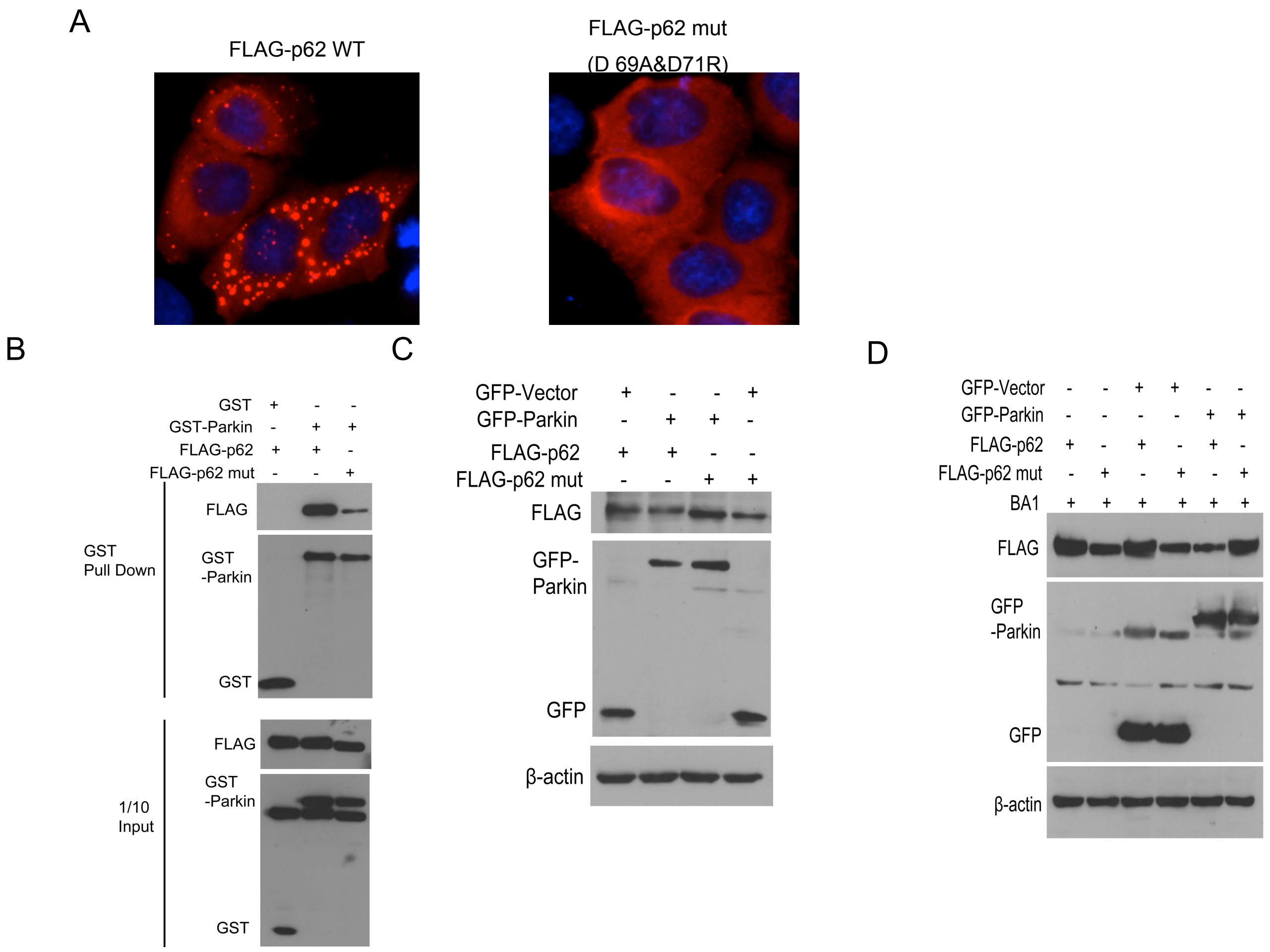

Figure S5. Parkin mediated polymerized p62 protein degradation.

A. Immunohistochemical analysis of p62 and p62 mutants (D69A & D71R) in HeLa cells.

B. Analysis the interaction of p62 with parkin; 293T cells were co-transfected GFP-parkin with FLAG-p62 or its mutants, and immunoprecipitation and western blotting were performed to examine the interaction between p62 and parkin.

C. 293T cells were transfected with GFP-parkin or GFP (control) with and p62 and p62 mutants for 24h, and then western blotting were performed to examine the p62 protein levels.

D. Described in Fig. S5c, before the cells were collected, the 20 nM of BA1 were added and furthermore examine the p62 protein levels.
